# An Interferon Response Gene Signature Is Associated with the Therapeutic Response of Hepatitis C Patients

**DOI:** 10.1371/journal.pone.0104202

**Published:** 2014-08-11

**Authors:** Lawrence M. Pfeffer, Kui Li, Jaquelyn F. Fleckenstein, Tony N. Marion, Joel Diament, Chuan He Yang, Susan R. Pfeffer, Meiyun Fan, Elizabeth Handorf, Charles R. Handorf

**Affiliations:** 1 Department of Pathology and Laboratory Medicine, and the Center for Cancer Research, University of Tennessee Health Science Center, Memphis, Tennessee, United States of America; 2 Department of Microbiology, Immunology and Biochemistry, University of Tennessee Health Science Center, Memphis, Tennessee, United States of America; 3 The Department of Medicine, Division of Gastroenterology, Washington University School of Medicine, St. Louis, Missouri, United States of America; 4 Biostatistics and Bioinformatics Facility, Fox Chase Cancer Center, Philadelphia, Pennsylvania, United States of America; University of Montreal Hospital Research Center (CRCHUM), Canada

## Abstract

Infection with the hepatitis C virus (HCV) is a major cause of chronic liver diseases and hepatocellular carcinoma worldwide, and thus represents a significant public health problem. The type I interferon (IFN), IFNα, has been successful in treating HCV-infected patients, but current IFN-based treatment regimens for HCV have suboptimal efficacy, and relatively little is known about why IFN therapy eliminates the virus in some patients but not in others. Therefore, it is critical to understand the basic mechanisms that underlie the therapeutic resistance to IFN action in HCV-infected individuals, and there is an urgent need to identify those patients most likely to respond to IFN therapy for HCV. To characterize the response of HCV-infected patients to treatment with IFNα, the expression of an IFN-response gene signature comprised of IFN-stimulated genes and genes that play an important role in the innate immune response was examined in liver biopsies from HCV-infected patients enrolled in a clinical trial. In the present study we found that the expression of a subset of IFN-response genes was dysregulated in liver biopsy samples from nonresponsive hepatitis C patients as compared with virologic responders. Based on these findings, a statistical model was developed to help predict the response of patients to IFN therapy, and compared to results obtained to the IL28 mutation model, which is highly predictive of the response to IFN-based therapy in HCV-infected patients. We found that a model incorporating gene expression data can improve predictions of IFN responsiveness compared to IL28 mutation status alone.

## Introduction

Hepatitis C virus (HCV) is estimated to infect ∼170 million people worldwide, causing a wide spectrum of liver diseases that varies from the asymptomatic carrier state to end-stage liver diseases [1]. These include chronic hepatitis, cirrhosis, liver failure, and hepatocellular carcinoma. Classified within the Flaviviridae family of enveloped, single-stranded, positive-sense RNA viruses, HCV has a tightly restricted host range confined to humans and chimpanzees, and replicates predominantly in hepatocytes. For reasons that have remained elusive, only a fraction of HCV-infected individuals spontaneously clear the virus, while the majority of HCV-infected individuals (70–80%) develop a chronic infection. Several structural and nonstructural proteins of HCV have been shown to antagonize the host innate immune response that is normally triggered by viral infection [2]. Viral RNA is a potent inducer of the host immune response and is recognized by specific Toll-like receptors in endosomal compartments or by the RNA helicases RIG-I and MDA5 in cytoplasm [3]. Rapid induction of the interferon (IFN) system, type I IFNs (IFNα, IFNβ and IFNω, type II IFN (IFNγ) and type III IFN (IL29, IL28A and IL28B), is a central event in establishing the host innate antiviral response that is downstream of TLR (Toll-like receptor)-dependent and TLR–independent pathways [4]. IFN acts in a paracrine and autocrine fashion to regulate gene expression that results in the induction of an antiviral state [5]. HCV control of the innate antiviral responses, especially at the level of IFN production, may provide a cellular foundation for viral persistence [6].

The pegylated derivative of IFNα (peg-IFN) and the antiviral drug ribavirin combined with a protease inhibitor is the current standard-of-care for HCV-infected patients [7]. IFNα has antiviral activity against a diverse variety of RNA and DNA viruses. When IFNα has been utilized as a monotherapy in chronically infected HCV patients, the success rate is ∼20%. Peg-IFNα, which has an improved half-life over standard IFNα, appears to have a somewhat higher success rate. However, it is unknown why IFNα therapy causes a sustained virological response in only a fraction of the patient population, as determined by the clearance of HCV. Moreover, several studies have identified specific cohorts of patients that have a relatively low response to these therapeutic regimens. For example, several studies established that the response rate of African-Americans is significantly lower than non-Hispanic whites [8]–[10]. This finding is of major health concern in the United States, since African-Americans account for ∼22% of the HCV-infected patients. Recently, single nucleotide polymorphisms within the IL28B locus, which encodes members of the IFNλ family, have been found to be highly predictive of the response to IFN/ribavirin therapy in HCV-infected patients [11]–[15]. While this represents a major advance in the field, the underlying mechanism for differential response in HCV-infected patients remains elusive.

To characterize the response of HCV-infected patients to treatment with type I IFN, the expression of an IFN-response gene signature was examined in liver biopsies. Based on microarray analysis we previously performed on IFN-treated human and mouse cells as well as a public database of IFN-stimulated genes (ISGs) [16]–[19], ISGs were stratified into genes that are most commonly induced by IFN and had putative binding sites with strong DNA binding scores for STAT2 (ISRE), STAT3/STAT1 (SIE), and NF-κB. The IFN-induced expression of these ISGs in Huh7 hepatoma cells was verified by quantitative real time PCR as being strongly IFN-induced (>10-fold) and the ISGs were placed into the following categories: 1) ISGs whose expression is predominantly driven by an ISRE; 2) ISGs whose expression is predominantly driven by an ISRE and potentially regulated by NF-κB; 3) ISGs whose expression is predominantly driven by an SIE; and 4) ISGs whose expression is predominantly driven by an SIE, and potentially regulated by NF-κB. An IFN signature gene list of 39 genes was then established based on a bioinformatic search of these genes as potentially playing important roles in the biological actions of IFN. The expression of this gene signature was examined in samples of formalin-fixed paraffin embedded (FFPE) tissue from liver biopsies obtained prior to the onset of any therapy in patients whose clinical course and response to IFN/ribavirin therapy was subsequently characterized in a clinical trial at UTHSC. Our results suggest that only a subset of the IFN-response gene signature was dysregulated in liver biopsies from chronically infected HCV patients. Moreover, we determined the predictive value of this gene signature and that of polymorphisms of IL-28 locus. Our results indicate that, besides nucleotide polymorphisms of IL-28 locus, this IFN response gene signature has important predictive value in determining which patients will most likely fail to respond to standard IFN-based hepatitis C therapy.

## Materials and Methods

### Patient Selection

Adult African American (AA) and Caucasian (C) patients with compensated chronic HCV were enrolled and treated at the UTHSC General Clinical Research Center as part of our clinical trial entitled “Racial Differences in HCV-Host Interaction”. Patients were required to be adult, AA or C with genotype 1A or 1B chronic hepatitis C with positive HCV RNA and no prior attempt at treatment. A liver biopsy was required with the histological diagnosis of chronic hepatitis. Patients with histological diagnosis of cirrhosis were enrolled if they did not have symptomatic portal hypertension and if they had a neutrophil count greater than 1500/mm^3^, platelet count of 85,000/mm^3^, albumin level greater than 3.0 g/dL and serum creatinine less than 1.4 mg/dL. All clinical investigation was conducted according to the principles expressed in the Declaration of Helsinki. All patients signed written informed consent specific for this protocol that was approved by the UTHSC IRB before entry into the study. Exclusion criteria included any cause of chronic liver disease other than HCV, HIV infection, active hemolytic anemia, evidence of decompensated cirrhosis with ascites, bleeding varices or portosystemic encephalopathy. In addition, patients with any known preexisting medical conditions that could interfere with participation such as uncontrolled seizure disorders, poorly controlled diabetes, serious pulmonary disease, immunologically-mediated disease, gout or any medical condition likely to require steroids during the course of the study were excluded from this study. Patients with cardiac ischemia, significant arrhythmia, cardiac failure, active substance abuse, retinal abnormalities, organ transplantation, HIV infection or serious psychiatric disease were also excluded. General clinical patient information is shown in Table 1.

**Table 1:** Enrolled patient information.

	AA	C
Patients enrolled	145	82
Male	54	38
Average age (s.d.)	48.5 (7.97)	44.3 (9.51)
Average weight^1^ (s.d.)	198.6 (32.91)	205.0 (39.76)
Female	91	44
Average age (s.d.)	48.9 (42.1)	46.0 (7.99)
Average weight^1^ (s.d.)	185.2 (41.92)	170.9 (56.18)
*Il28B* rs12979860 Genotype^2^	CC 9 (8.4%)	20 (33%)
	TT 39 (36%)	6 (10%)
	C/T 59 (55%)	34 (57%)
Infected with genotype 1a^3^	99	69
1b	45	10
Average years infected (max/min)	22 (43/1)	21 (47/3)
Average pre-therapy serum HCV IU/ml^4^ (s.d.)	1,064,878 (1,377,098)	1,599,894 (2,451,571)
Average ALT (s.d.)	88 (62)	103 (65)
Average fibrosis score (s.d.)^5^	2.0 (1.1)	1.9 (0.9)
Mild fibrosis (0–2)	104	56
Advanced fibrosis (3–4)	39	26

1 Weight is in US pounds.

2 Likelihood ratio = 24.72, *p = 4.3×10^−6^*. *Il28B* rs12979860 SNP genotypes were determined for 107 AA and 60 C.

### Treatment Regimen

All patients were treated with standard weight-based therapy with 1.5 mg/kg of pegylated IFNα2b subcutaneously once per week and 13 mg/kg of ribavirin PO daily for up to 48 weeks. Therapy was discontinued after 24 weeks if patients did not have a negative HCV RNA level. Use of erythropoietin was allowed.

Patients who completed at least 12 weeks of therapy were classified as Sustained Viral Responders (SVR) if they cleared virus on treatment and remained virus-free for 6 months after completion of therapy. Non–responders (NR) were defined as patients who never cleared virus. Patients who initially had a negative HCV RNA level upon treatment but subsequently became positive again for HCV RNA (either during continued treatment or within 6 months after completing therapy) were defined as Relapsers (R).

### Gene expression analysis of RNA obtained from FFPE liver biopsies

Formalin-fixed paraffin embedded (FFPE) pre-treatment liver biopsy specimens from ∼130 patients were retrieved, 3–5 (5 µm) curls were cut from each liver biopsy and RNA isolated using the RecoverAll Total Nucleic Acid Isolation Kit (Ambion Inc.) according to the manufacturer's directions. Gene expression analysis was conducted on the nCounter Analysis System (NanoString Technologies) using a codeset designed to target 43 genes (39 genes in an IFN gene signature and 4 potential house-keeping genes: ACTB, GAPDH, GUSB, PGK1) and 15 controls (58-plex codeset). The 39 genes in the IFN gene signature targeted in the gene array are listed in Table 2. In brief, 100 ng of total RNA was mixed with pairs of capture and reporter probes and hybridized on the nCounter Prep Station. Upon removal of unbound probes, the purified ternary complexes were bound to the imaging surface, elongated, immobilized, and quantified on the nCounter digital analyzer. To account for differences in hybridization and purification, data were normalized to the average counts for all control spikes in each sample and analyzed with nSolver software. The expression data was normalized by using a geometric mean of the four housekeeping genes (ACTB, GAPDH, GUSB, PGK1) in our codeset as previously described [20]. Only two RNA samples out of the 132 samples prepared from FFPE liver biopsies were not used in further analysis due to low retrieved RNA levels or marked degradation of RNA extracted from the FFPE tissue. Thus, gene expression in 130 samples was subjected to analysis and represented 43 hepatitis C-infected patients who responded to IFNα-ribavirin therapy (responders), 56 hepatitis C-infected patients who did not respond to therapy (nonresponders), and 31 patients who had an initial virological response but then relapsed and exhibited high viral HCV titers (relapsers). FFPE tissue from 80 African-American and 50 Caucasian hepatitis C-infected patients were studied in this dataset.

**Table 2:** Expression of the IFN Gene Signature.

Gene	Minimum	Maximum	Mean	Std Error	Median
ANGPT2 ^b^	1	132.8	7. 7	1.4	2.3
CASP4 ^c^	54.4	214.6	102.9	2.8	95.4
CCL5 ^b^	49.1	469.2	160.5	6.6	143.6
DDX58 ^b^	60.7	450.9	200.8	7.3	189.7
DHX58 ^a^	84.9	507.1	223.7	8.1	215.4
EIF2AK2 ^a^	117.6	554.1	311.7	9.6	309.3
GADD45B ^d^	56.6	584.2	158.5	6.9	144.6
GADD45G ^d^	1	112.6	16.9	1.7	12.3
GBP1 ^b^	94.8	482.9	266.2	7.38	260.4
HIF3A ^d^	1	202.2	8.6	2.5	1
IFI16 ^a^	43.3	280.1	132.5	4.5	125.6
IFI6 ^a^	258.6	11402.8	5904.4	289.9	6385.7
IFIH1 ^b^	51.6	316.4	158.4	5.9	157
IFNB1 ^b^	1	274	5.8	2.5	1
IL6 ^b^	1	79.2	6.9	1.4	1
IRF1 ^d^	27.9	208.7	78.8	3.1	69.5
IRF2 ^a^	95.2	422.7	188.8	4.4	184
IRF5 ^b^	10.8	253.8	45.9	2.9	39
IRF7 ^b^	107.5	1121	323.8	15.08	302.5
IRF9 ^b^	293.3	1524.4	635.5	17.0	631
ISG20 ^b^	23.8	395	111.8	6.0	97. 7
MAVS ^c^	108	939.5	282.6	10.8	261.6
MX1 ^a^	1	96.8	24.7	2.0	20.1
MYD88 ^d^	107	378.6	217.2	4.8	219.3
OAS1 ^a^	198.5	1660.4	905.5	35.7	932.2
PLSCR1 ^a^	96.5	560	248.2	8.5	244.8
PML ^a^	49.1	798.3	207.5	10.7	184.1
RARRES3 ^a^	200	797.6	405.0	11.0	393.7
RSAD2 ^a^	1.6	346.5	118.3	7.4	108.6
SOCS1 ^a^	1	99.6	10.4	1.4	5.1
STAT1 ^b^	347.8	3343.8	1492.0	53.2	1456.6
STAT2 ^a^	413	1532.1	818.0	21.0	816.3
TAP1 ^a^	28.1	253.8	86.8	3.2	84.6
TLR2 ^a^	21.3	213.5	59.5	3.2	51.3
TLR3 ^a^	4.2	193.3	45.9	2.3	43.1
TNFRSF10B ^c^	33.7	194.8	77.5	2.7	72.8
TNFSF10 ^a^	303.1	1929.8	898.1	29.7	857
VEGFC ^c^	1	92.4	17.9	1.6	12.4
XAF1 ^d^	101.9	988	403.9	15.6	421.5

**Key:** ^a,^ISRE-driven ISGs, ^b^ ISRE-driven ISGs potentially regulated by NF-κB, ^c^ SIE-driven ISGs, and ^d^ SIE-driven ISGs potentially regulated by NF-κB.

### IL28B SNP sequence analysis

The 184 bp genomic DNA fragment that included the *19q13* rs12979860 SNP at nucleotide 92 was amplified by PCR using the following primers: forward 5′-CTGCACAGTCTGGGATTCC-3′ and reverse 5′-TCACAGAAGGGAGCCCTGC-3′. Nucleotide sequences were generated with an ABI Model 3130XL Gene Analyzer (Applied Biosystems, Life Technologies, Carlsbad, CA) in the UTHSC Molecular Resource Center, and the sequence at position 92 of the PCR fragment was determined using Sequence Scanner v.1.0 (Applied Biosystems).

### Statistical analysis and modeling

The data on expression of the IFN gene signature in the different patient subgroups were subjected to nonparametric Mann-Whitney analysis using Graphpad InStat 3 software, with p-values <0.05 considered statistically significant. We then built a predictive model for response using logistic regression. Only full responders were considered successfully treated, with relapsers and non-responders considered unsuccessful. In our initial model, self-reported race and continuous expression of each statistically significant genes identified in univariable analysis were included as predictors of response to interferon. We then refined our model using a backwards selection procedure, where the model was fit repeatedly, removing the least informative genes at each step. Patients with a model-based probability of >0.5 were categorized as predicted responders. We used 10-fold cross validation to test the predictive ability of each model [21], so that our final model demonstrated the highest agreement with true response across the validation sets. We also considered models in the subset of patients whose IL28B SNP data was available. Under a per-allele model, we added IL28B status to the logistic regression models and compared the results to expression-based models. The regression procedures were performed using R software (version 2.13).

## Results

### The expression of an IFN-response gene signature in liver biopsies

Total RNA was extracted from curls cut from FFPE liver biopsies collected from patients prior to the initiation of therapy, and assayed on a Nanostring nCounter for expression of an IFN gene signature that we designed based on our own microarray results, databases of IFN-stimulated genes (ISGs), and regulatory elements in gene promoter regions. The expression of these genes was then normalized to the expression of four housekeeping genes (Actin B, GAPDH, GUSB and PGK1) that was included in the Nanostring analysis. As shown in Table 1 there was a large sample-to-sample variation in the expression of the genes in the IFN signature. Some genes were expressed at low but detectable levels such as cytokine and growth factors, including ANGPT2, IFNB1, IL6 and VEGFC, as well as classical IFN-stimulated genes such as Mx1, SOCS1 and TLR3. In addition a number of genes critical in the IFN response pathway, which included GBP1, MAVS, TNFSF10 (TRAIL), and XAF1, were expressed at relatively high levels in liver biopsies. These results are consistent with our previous finding that low levels of type I IFNs could be detected in the sera of patients chronically infected with HCV [22], as well as the findings that ISG expression could be detected in the livers of HCV-infected patients and in experimentally-infected chimpanzees [23]–[26].

### The expression of a subset of the IFN gene signature correlates with the patient response to therapy

**Figure 1:**
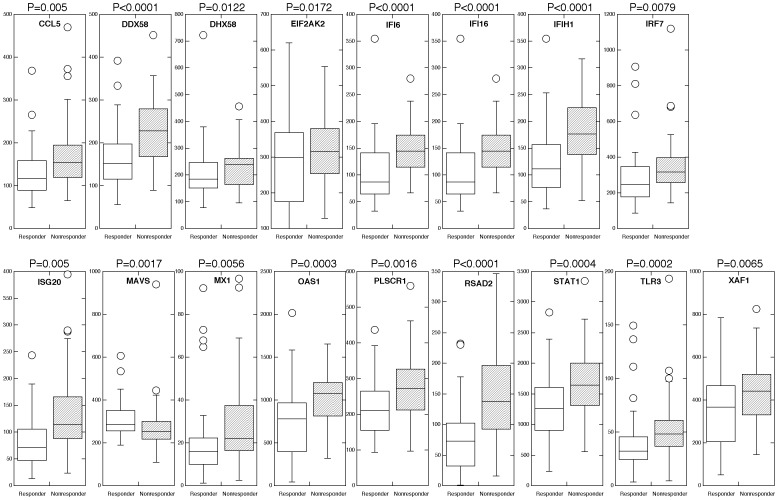
Differential expression of an IFN-regulated signature geneset in responders versus nonresponders to therapy. Expression of an IFN-regulated signature geneset was determined in RNA extracted from FFPE liver biopsies by nCounter analysis. Boxplots of genes found to be statistically differentially expressed by nonparametric Mann-Whitney analysis (p<0.05).

In the 130 liver samples subjected to Nanostring analysis, 43 were obtained from HCV-infected patients who responded to IFN-ribavirin therapy (responders), 56 from HCV-infected patients who did not respond to therapy (nonresponders), and 31 from patients who had an initial virological response to therapy, but then relapsed during therapy and had high viral HCV titers (relapsers). Of these 130 patient samples, 112 had their IL28B genotype determined (Table 3). The liver tissue was obtained from 80 African-American (AA) and 50 Caucasian hepatitis C-infected patients (Table 4). Consistent with the previous findings that African Americans demonstrate a lower response rate to IFN than Caucasians, in the African American group of patients 27.5% were classified as responders, 57.5% as non-responders and 15% as relapsers. In contrast, in the Caucasian group of patients 50% were classified as responders, 22.1% as non-responders and 27.9% as relapsers. Initial analysis of differences in gene expression between responders and non-responders (excluding relapsers) revealed statistically significant differences (p<0.05) in the expression of 16 out of the 39 genes examined, which included CCL5, DDX58/RIG-I, DHX58/LGP2, EIF2AK2, IFI6, IFI16, IFIH1/MDA5, IRF7, ISG20, MX1, OAS1, PLSCR1, RSAD2, STAT1, TLR3 and XAF1 (Figure 1). Most interestingly, these genes were expressed at higher levels in liver biopsies from patients that did not respond to IFN-ribavirin therapy as compared to the responders to therapy. The higher expression levels of ISGs prior to therapy in nonresponders compared with responders was consistent with several previous reports [27]–[30]. It is important to note that there was no relationship found between the expression of the IFNβ gene in liver biopsies, and responsiveness to exogenous IFN therapy. This finding is consistent with our previous report that serum levels of type I IFNs in patients chronically infected with HCV did not correlate with the response to exogenous IFN therapy [22].

**Table 3:** IL28 Genotype analysis (112/130 patients).

Genotype		R	NR	Relapser		AA	CA
CT		21 (35%)	22 (37%)	17 (28%)		35 (58%)	25 (52%)
TT		4 (11%)	21 (60%)	10 (29%)		29 (83%)	6 (17%)
CC		10 (59%)	3 (18%)	4 (23%)		5 (29%)	12 (71%)

**Table 4:** Patient Demographics.

Patient Response	AA	Caucasians
Responders	22 (17%)	25 (19%)
Nonresponders	46 (35%)	11 (9%)
Relapsers	12 (9%)	14 (11%)

**Figure 2:**
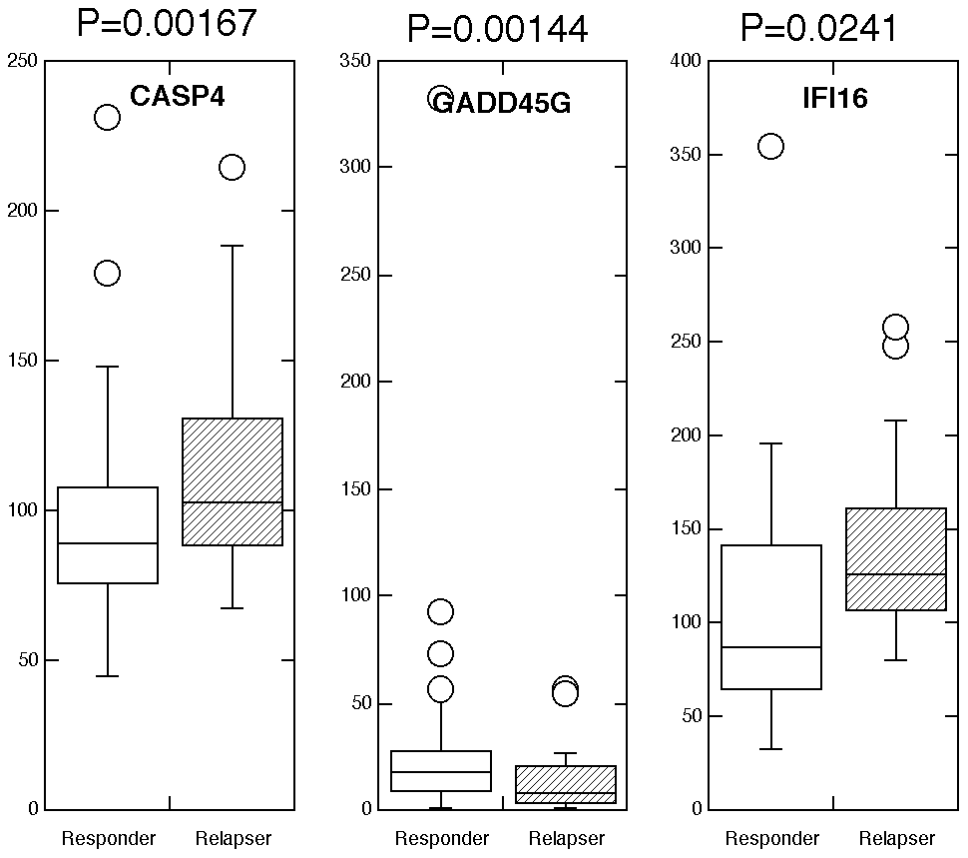
Differential expression of an IFN-regulated signature geneset in relapsers versus responders to IFN-ribavirin therapy to therapy. Expression of an IFN signature geneset was determined in RNA extracted from FFPE liver biopsies by nCounter analysis. Boxplots of genes found to be statistically differentially expressed by nonparametric Mann-Whitney analysis (p<0.05).

In addition we subjected the data collected on gene expression from relapsers and compared it to the data from responders and nonresponders. Although there was no statistical difference between the various genes in nonresponders and relapsers, the expression of CASP4, GADD45G and IFI16 was found to be statistically different between the relapsers and the responders to IFN-ribavirin therapy. As shown in Figure 2, while CASP4 and IFI16 expression was higher in the group of relapsers, GADD45G expression was found to be somewhat lower. In addition, we compared the expression of the IFN gene signature according to race and found no statistical differences according to race, i.e. AA responders did not differ from Caucasian responders, etc.

### Cellular functions of genes associated with IFN responsiveness

**Figure 3:**
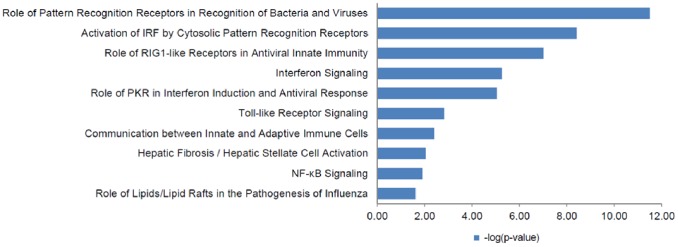
Signaling networks of genes that are associated with IFN responsiveness. The gene network was generated using Ingenuity Pathway Analysis software.

**Figure 4:**
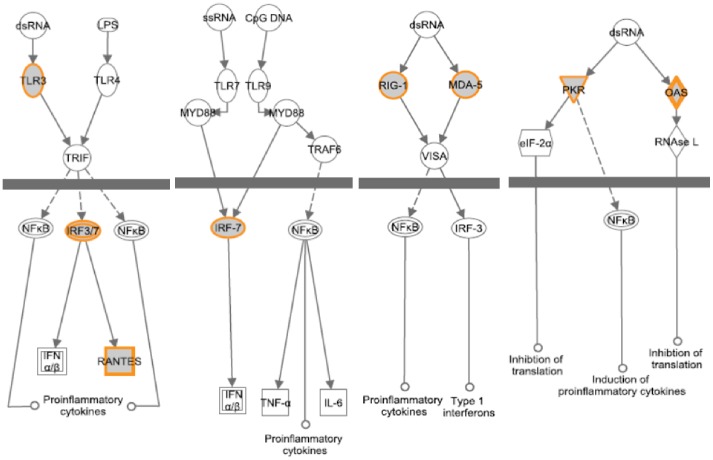
Genes in the pattern recognition receptor pathway are associated with IFN responsiveness. The gene network was generated using Ingenuity Pathway Analysis software. Genes upregulated in nonresponders (shown in Figure 1) were highlighted. As expected, several antiviral signaling pathways were overrepresented in these genes.

**Figure 5:**
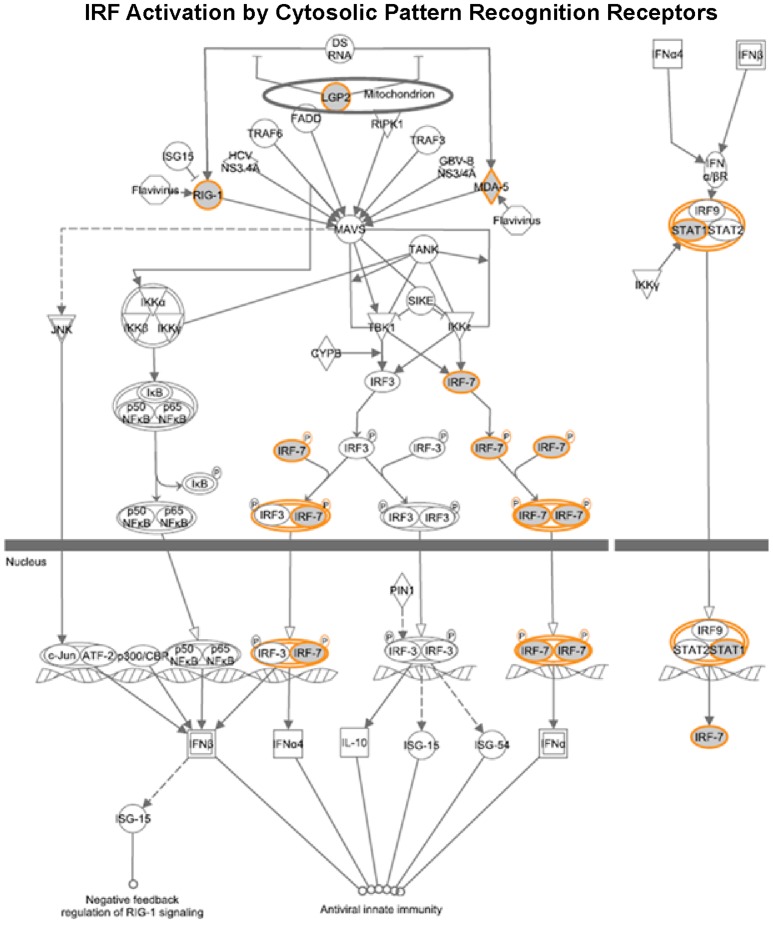
Genes in IRF activation by cytosolic pattern recognition receptors are associated with IFN responsiveness. The gene network was generated using Ingenuity Pathway Analysis software. Genes upregulated in nonresponders (shown in Figure 1 and 2) were highlighted.

To gain insights into the cellular functions of the genes that were found associated with IFN responsiveness, we performed signaling pathway analysis using the Ingenuity Pathway analysis (IPA) software. As shown in Figure 3, 4 and 5, several critical antiviral signaling pathways were overrepresented in the genes that are associated with IFN responsiveness, For example TLR3, RIG-I/DDX58, MDA5/IFIH1, PKR/EIF2AK2, OAS1, IRF7 and RANTES/CCL5 are expressed at higher levels in nonresponders and are components in the pattern recognition receptor (PRR) pathway that is involved in the recognition of bacteria and viruses (Figure 4). Of these, TLR3 is known to recognize dsRNA intermediates produced during HCV RNA replication [31], [32], while RIG-I senses 5′-triphosphate-bearing, genomic or antigenomic HCV RNAs [33]. Activation of either pathway in hepatocytes leads to an IFN response that restricts HCV replication. PKR has also been shown to recognize HCV RNA [34], although it is uncertain whether PKR has an antiviral and/or proviral effect [2]. OAS1 is a component of the OAS-RNase L system that had been shown to detect and degrade HCV RNA in HeLa cytoplasmic extracts [35]. In addition, RIG-I, LGP2, MDA5, IRF7 and STAT1 are also expressed at higher levels in nonresponders (Figure 5) and are components of the pathways leading to IRF activation by cytosolic PRRs or signaling to ISG induction downstream of the IFN receptors, which result in an innate immune response against viruses and bacteria. Furthermore, genes with significantly higher expression in nonresponders included two ISGs that possess anti-HCV activity in cell culture, i.e. RSAD2/viperin and ISG20 [24], [36].

### Nucleotide polymorphism in the IL28B gene is highly predictive of the response to IFN/ribavirin in HCV-infected patients

AA in the present study had ∼three-fold higher inheritance of the homozygous *TT* genotype at the *Il28B*-associated rs12979860 SNP compared to homozygous *CC* (Table 3). In contrast, C had ∼four-fold higher inheritance of *CC* compared to *TT*. These results are consistent with the well-established racially associated difference in inheritance at rs12979860 in chronic HCV infection [11]–[14]. The inheritance of the CC versus TT or CT genotypes at rs12979860 was highly correlated with (OR = 3.17, 95% CI 1.004 – 9.989, *p = 0.049*) a sustained response of HCV-infected patients to IFN-ribavirin therapy (responders). In addition, failure to respond to therapy (nonresponders), or an initial virological response to therapy but then relapsed with high viral HCV titers (relapsers) was highly dependent upon rs12979860 genotype (*p = 0.000972*) and race (*p = 0.00861*). Although rs12979860 genotype and other *il28B*-associated SNPs in linkage disequilibrium may be the most important correlates for racially associated differences in response to IFN-ribavirin therapy for hepatitis C, factors not associated with the *il28B* locus also contribute to racially associated response differences.

### The development of a predictive model for the patient response to therapy based on the expression of IFN signature genes, and nucleotide polymorphism in the IL28B gene

**Figure 6:**
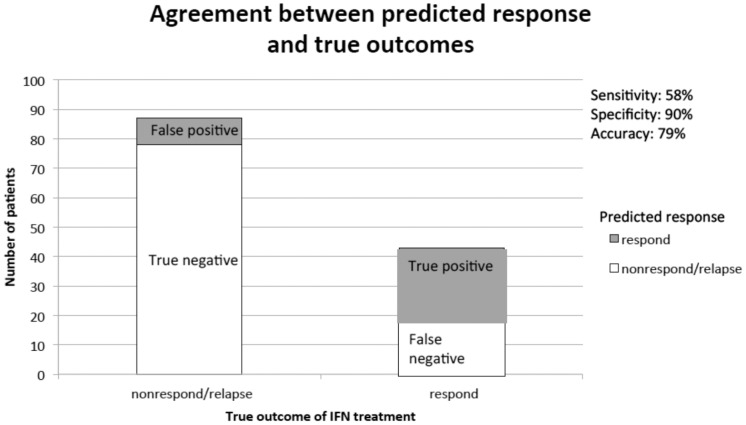
The distribution of predicted probability of therapeutic response (with relapsers included). We found that those who actually responded to IFN-ribavirin treatment had higher predicted probabilities of response in the model.

Using logistic regression with cross validation, we found that the best model contained RSAD2, IFI6, IFI16, STAT1, CCL5, and XAF1. We show the results of the logistic regression models in Table 5. We note that not all variables were statistically significant in the final models. Although we used statistical significance to help develop candidate models, we selected the final model based on its predictive potential in the 10-fold cross validation procedure. Higher expression levels of RSAD2, IFI6, IFI16, and CCL5 were associated with a reduced probability of patient response to IFN. Lower expression of STAT1 and XAF1 were associated with increased probability of response. Interestingly, race was not strongly predictive of response when controlling for the effects of these genes, indicating that we may have identified some of the genetic factors that reduce response rates among African Americans. Our model accurately predicted response for 79.2% of individuals, with a specificity of 90% and sensitivity of 58% (See Figure 6). Therefore, although the model rarely predicted response when the true outcome was nonresponse/relapse (false positives), it lacked sensitivity with several false negatives. Model-based predictions were superior to race alone, which was not very predictive of response with only 60% overall accuracy.

**Table 5:** Logistic regression modeling of patient data.

Expression-based model (N = 130)			
Gene	OR*	95% CI	
RSAD2	0.863	0.755	0.986
IFI6	0.997	0.994	0.999
IFI16	0.867	0.758	0.992
STAT1	1.014	1.000	1.029
CCL5	0.927	0.857	1.002
XAF1	1.053	1.008	1.100

Expression and IL28 model (N = 115)			
Gene	OR*	95% CI	
RSAD2	0.947	0.850	1.056
IFI6	1.000	0.997	1.002
IFI16	0.978	0.869	1.101
CCL5	0.910	0.833	0.993
IL28B	2.988	1.271	7.024

*OR for gene expression is for a change of 10 units.

IL28B status was available in 112 out of the 130 patients whose IFN-response gene signature was examined. In this subset of patients, our expression-based model using RSAD2, IFI6, IFI16, CCL5, XAF1, and STAT1 was 73.0% accurate. Although this was lower than accuracy for the cohort as a whole, it still performed better than other expression-based models. We found that we could improve performance by including IL28B status. Combining this with RSAD2, IFI6, IFI16, and CCL5 had 74.9% accuracy. Importantly, this model had improved accuracy over a model with IL28B and race alone (without expression data), which had an accuracy of 67.8%.

## Discussion

Approximately 3% of the world's population is infected with HCV, and ∼80% of HCV-infected individuals eventually become chronically infected. While long-term persistence results predominantly from evasion of the adaptive immune response to viral infection, evasion of host innate immune response is believed to contribute to establishing persistent HCV infection [6]. The IFN system is a key player in the innate immune response against viral infections, by inducing an antiviral state in the host against a variety of viral pathogens. Moreover, HCV is highly sensitive to treatment with type I and type III IFNs in vitro, and IFN remains a mainstay of treatment of HCV infected patients [37]. However, precisely why the current IFNα-based therapy is only effective in a proportion of treated patients remains largely a mystery, and this has been an area of intensive investigation. In the present study we compared expression of an IFN-response geneset in FFPE derived from liver biopsies from patients enrolled in an IFN-ribavirin trial conduced at UTHSC, which were taken before therapy was initiated. Our goal was to establish an IFN-response gene signature that can be used to predict IFN-responsiveness in hepatitis C patients.

The IFN-response signature geneset was based on our own microarray studies on IFN-induced genes in human and mouse cell lines, a database of IFN-stimulated genes from a variety of cells, and regulatory elements of gene promoter regions. Our strategy was to focus on genes that may play an important role in the antiviral innate immune response, and that were induced by various IFN activated signaling pathways. Thus genes such as Mx1 and PKR/EIF2AK2 contain an ISRE (IFN-stimulated response element), and are induced by the classical IFN-activated signaling that involved JAK1 and TYK2 activation and STAT1 and STAT2 transcription factors [38]. However ISGs such as RIG-I/DDX58 and MDA5/IFIH1 may be induced through an NF-κB-dependent pathway as well [16]. In addition, a number of ISGs such as VISA/MAVS and Casp4 do not contain an ISRE but rather an SIE (c-sis-inducible element) and appear to be regulated by STAT1 and STAT3 homodimers and heterodimers [39]. In addition, some SIE-containing ISGs such as IRF1 and MYD88 may also be regulated through an NF-κB-dependent pathway [18]. All 39 members of the IFN-regulated signature gene set were induced upon IFNα treatment of Huh7 hepatoma cells (data not shown).

We found that nearly all 39 of the IFN-response signature genes were expressed in liver biopsies from chronically HCV-infected patients enrolled in the clinical trial of IFN-ribavirin at UTHSC. Previous studies using microarrays to determine global gene expression of liver biopsies from chronically HCV-infected patients and from experimentally HCV-infected chimpanzees show elevated ISG expression [23]–[26]. In addition, there was a great deal of heterogeneity in the expression level of the signature genes in the different patients. For example, genes that were expressed at relatively low levels such as IFNβ, IL6, TNFRSF10B, VEGFC and ANGPT2 were expressed only in a subset of patient samples. In contrast, genes that were expressed at relatively high levels such as TNFSF10 (TRAIL), OAS1, RANTES (CCL5) and IRF9 were detected in all liver biopsies. It is of particular interest that only a small fraction of liver biopsies (∼10%) from patients chronically infected with HCV expressed the IFNβ gene. While this seems to conflict with our previous finding that ∼50% of the serum samples from a smaller patient population in the clinical trial of IFN-ribavirin had detectable levels of type I IFN [22], it is conceivable that most of the circulating levels of type I IFN derive from extrahepatic sources such as the plasmacytoid dendritic cells. Consistent with our data, two recent studies have shown that type III IFNs rather than type I IFNs are induced in the liver of experimentally HCV-infected chimpanzees and that the intrahepatic level of type III IFNs correlates with that of ISG expression [40], [41]. Our IFN-response geneset did not contain type III IFN genes, since when this study was initiated and the geneset was constructed a role for IL29/IL28B in hepatic innate immunity to HCV infection was not recognized. Nevertheless, it is worth noting that intrahepatic type III IFN level was not found to associate with the outcome of acute HCV infection [40], nor was it related to response to IFN therapy in patients with chronic hepatitis C [42].

Another important finding from these studies is that expression of a subset of these genes in liver biopsies inversely correlated with the responsiveness to IFN-ribavirin therapy, i.e. higher expression was observed in nonresponders as compared to responders. Although this finding is counterintuitive, as one would expect an active IFN system would help eliminate the virus during therapy, it is supported by a number of studies, which have shown that patients with a high ISG expression prior to the initiation of IFN therapy seem to respond poorly to IFN therapy [27]–[30]. Additional support is lent from a study showing that the expression of intrahepatic ISGs was already maximally induced in chimpanzees chronically infected with HCV. Consequently, when exogenous IFN was administered, there was no further ISG upregulation [43]. Although the underlying mechanism remains elusive, nonresponder hepatitis C patients tend to have pre-activated Jak-STAT pathway prior to therapy, which may connect to IFN refractoriness [27].

However, it is important to note that in our study only a subset of the IFN-regulated genes examined were expressed at a statistically significant higher level in patients that were nonresponsive to IFN therapy. Of particular interest, some of these overexpressed genes were either involved in viral sensing (TLR3, RIG-I/DDX58, DHX58/LGP2, MDA5) or effector functions of IFNs (ISG20, RSAD2, PKR, etc.). Both TLR3 and RIG-I can sense HCV RNA (although they recognize different viral ligand forms) early after infection and initiate signaling pathways culminating in the induction of an IFN response that curtails HCV replication [32], [33]. However, HCV has evolved to disarm both mechanisms by NS3/4A-mediated cleavage of the essential adaptor proteins, TRIF (Toll/interleukin receptor domain-containing adapter-inducing interferon), and MAVS, once the infection is established in hepatocytes [44]–[46]. Among the ISG effectors, ISG20 and RSAD2 have been shown to inhibit HCV replication, while controversial results have been reported for PKR [2]. There are several possibilities that may explain why induction of the endogenous anti-viral ISGs prior to IFN therapy fails to contain HCV infection [47]. The anti-HCV ISGs may only be transcriptionally upregulated in uninfected surrounding cells but not in HCV-infected hepatocytes. Alternatively, ISG transcripts may be made in both infected and uninfected cells but ISG proteins are only made in uninfected cells because of PKR activation in infected cells [48]. Third, some HCV proteins may inhibit the effector functions of the antiviral ISGs. Regardless of the infection/treatment outcome, the induction of a subset of IFN-regulated signature genes we identified in treatment-naïve patient liver underscores the important roles these genes/pathways may play in host attempts to control HCV infection in the liver.

In addition, we found CCL5/RANTES to be over-expressed in both nonresponders and relapsers to IFN therapy. CCL5 is a chemokine produced by monocyte/macrophage subsets in the liver, which contributes to recruiting T cells and other leukocytes to the infection site and also to the progression and resolution of liver fibrosis [49]. A recent study from our group suggests that infected hepatocytes represent a cellular source for CCL5 production at early phase of HCV infection, through TLR3-mediated sensing of HCV dsRNA intermediates and subsequent activation of NF-κB, a transcription factor pivotal for RANTES synthesis [31]. CCL5 is associated with a Th1 lymphocyte-related cytokine/chemokine profile and HCV clearance [50]. However, CCL5 may also shape the IFN response in the liver by altering the infiltration and activation of hepatic stellate cells, which maintains chronic HCV infection in the liver in part by inhibiting liver fibrosis [51]. Exactly how pre-activated intrahepatic RANTES expression affects IFN responsiveness will require further study.

Our study also presents a predictive model for IFN responsiveness based on a small number of signature genes. Although variants of the IL28B/IFNλ3 gene have recently been found to be highly predictive of the response to IFN/ribavirin in HCV-infected patients [11]–[15], there has been a paucity of mechanistic insights. Clearly, intrahepatic type III IFN levels of hepatitis C patients are not affected by IL28B polymorphism [42], [52], nor is it the case in primary hepatocyte cultures infected by HCV in vitro [40], [53]. Thus, the underlying mechanism for differential response in HCV-infected patients remains largely unknown. In our study we show that the expression of RSAD2, IFI6, IFI16, STAT1, CCL5, and XAF1 was highly predictive of the eventual IFN responsiveness to IFN/ribavirin therapy. Future studies will elucidate why lower expression of STAT1 and XAF1 were predictive of IFN responsiveness while expression of RSAD2, IFI6, IFI16, and CCL5 were associated with a poorer response to therapy. The model must also be validated with external data, and though our model had high specificity, further refinement is needed to improve model sensitivity.

## References

[1] Lemon SM (2010) Induction and evasion of innate antiviral responses by hepatitis C virus. J Biol Chem 285: 22741–22747. 20457596 10.1074/jbc.R109.099556 PMC2906263

[2] Li K, Lemon SM (2013) Innate immune responses in hepatitis C virus infection. Semin Immunopathol 35: 53–72. 22868377 10.1007/s00281-012-0332-x PMC3732459

[3] Meylan E, Tschopp J, Karin M (2006) Intracellular pattern recognition receptors in the host response. Nature 442: 39–44. 16823444 10.1038/nature04946

[4] Lester SN, Li K (2014) Toll-Like Receptors in Antiviral Innate Immunity. J Mol Biol 426: 1246–1264. 24316048 10.1016/j.jmb.2013.11.024 PMC3943763

[5] Pfeffer LM, Dinarello CA, Herberman RB, Williams BR, Borden EC, et al (1998) Biological properties of recombinant alpha-interferons: 40th anniversary of the discovery of interferons. Cancer Res 58: 2489–2499. 9635566

[6] Gale M Jr, Foy EM (2005) Evasion of intracellular host defence by hepatitis C virus. Nature 436: 939–945. 16107833 10.1038/nature04078

[7] Ghany MG, Nelson DR, Strader DB, Thomas DL, Seeff LB (2011) An update on treatment of genotype 1 chronic hepatitis C virus infection: 2011 practice guideline by the American Association for the Study of Liver Diseases. Hepatology 54: 1433–1444. 21898493 10.1002/hep.24641 PMC3229841

[8] Fleckenstein J (2004) Chronic hepatitis C in African Americans and other minority groups. Curr Gastroenterol Rep 6: 66–70. 14720456 10.1007/s11894-004-0028-z

[9] Castellino S, Lensing S, Riely C, Rai SN, Davila R, et al (2004) The epidemiology of chronic hepatitis C infection in survivors of childhood cancer: an update of the St Jude Children's Research Hospital hepatitis C seropositive cohort. Blood 103: 2460–2466. 14684419 10.1182/blood-2003-07-2565

[10] Muir AJ, Bornstein JD, Killenberg PG (2004) Peginterferon alfa-2b and ribavirin for the treatment of chronic hepatitis C in blacks and non-Hispanic whites. N Engl J Med 350: 2265–2271. 15163776 10.1056/NEJMoa032502

[11] Ge D, Fellay J, Thompson AJ, Simon JS, Shianna KV, et al (2009) Genetic variation in IL28B predicts hepatitis C treatment-induced viral clearance. Nature 461: 399–401. 19684573 10.1038/nature08309

[12] Suppiah V, Moldovan M, Ahlenstiel G, Berg T, Weltman M, et al (2009) IL28B is associated with response to chronic hepatitis C interferon-alpha and ribavirin therapy. Nat Genet 41: 1100–1104. 19749758 10.1038/ng.447

[13] Tanaka Y, Nishida N, Sugiyama M, Kurosaki M, Matsuura K, et al (2009) Genome-wide association of IL28B with response to pegylated interferon-alpha and ribavirin therapy for chronic hepatitis C. Nat Genet 41: 1105–1109. 19749757 10.1038/ng.449

[14] Thomas DL, Thio CL, Martin MP, Qi Y, Ge D, et al (2009) Genetic variation in IL28B and spontaneous clearance of hepatitis C virus. Nature 461: 798–801. 19759533 10.1038/nature08463 PMC3172006

[15] Rauch A, Kutalik Z, Descombes P, Cai T, Di Iulio J, et al (2010) Genetic variation in IL28B is associated with chronic hepatitis C and treatment failure: a genome-wide association study. Gastroenterology 138: 1337–1345 e1331 – 10.1053/j.gastro.2009.12.056 20060832

[16] Pfeffer LM, Kim JG, Pfeffer SR, Carrigan DJ, Baker DP, et al (2004) The role of NF-κB in the antiviral action of interferon and interferon-regulated gene expression. J Biol Chem 279: 31304–31311. 15131130 10.1074/jbc.M308975200

[17] Wei L, Fan M, Xu L, Heinrich K, Berry MW, et al (2008) Bioinformatic analysis reveals cRel as a regulator of a subset of interferon-stimulated genes. J Interferon Cytokine Res 28: 541–551. 18715197 10.1089/jir.2007.0136 PMC2988468

[18] Wei L, Sandbulte MR, Thomas PG, Webby RJ, Homayouni R, et al (2006) NFκB negatively regulates interferon-induced gene expression and anti-influenza activity. J Biol Chem 281: 11678–11684. 16517601 10.1074/jbc.M513286200 PMC1457055

[19] Rusinova I, Forster S, Yu S, Kannan A, Masse M, et al (2013) Interferome v2.0: an updated database of annotated interferon-regulated genes. Nucleic Acids Res 41: D1040–1046. 23203888 10.1093/nar/gks1215 PMC3531205

[20] Vandesompele J, De Preter K, Pattyn F, Poppe B, Van Roy N, et al (2002) Accurate normalization of real-time quantitative RT-PCR data by geometric averaging of multiple internal control genes. Genome Biol 3 RESEARCH0034. 10.1186/gb-2002-3-7-research0034 PMC126239 12184808

[21] Hastie T, Tibishirani R, Friedman J (2009) Elements of Statistical Learning: Data Mining, Inference and Prediction (Second Edition). New York: Springer-Verlag.

[22] Pfeffer LM, Madey MA, Riely CA, Fleckenstein JF (2009) The Induction of Type I Interferon Production in Hepatitis C-Infected Patients. J Interferon Cytokine Res 29: 299–306. 19232000 10.1089/jir.2008.0092 PMC2956646

[23] Bigger CB, Brasky KM, Lanford RE (2001) DNA microarray analysis of chimpanzee liver during acute resolving hepatitis C virus infection. J Virol 75: 7059–7066. 11435586 10.1128/JVI.75.15.7059-7066.2001 PMC114434

[24] Helbig KJ, Lau DT, Semendric L, Harley HA, Beard MR (2005) Analysis of ISG expression in chronic hepatitis C identifies viperin as a potential antiviral effector. Hepatology 42: 702–710. 16108059 10.1002/hep.20844

[25] Lau DT, Fish PM, Sinha M, Owen DM, Lemon SM, et al (2008) Interferon regulatory factor-3 activation, hepatic interferon-stimulated gene expression, and immune cell infiltration in hepatitis C virus patients. Hepatology 47: 799–809. 18203148 10.1002/hep.22076

[26] Su AI, Pezacki JP, Wodicka L, Brideau AD, Supekova L, et al (2002) Genomic analysis of the host response to hepatitis C virus infection. Proc Natl Acad Sci U S A 99: 15669–15674. 12441396 10.1073/pnas.202608199 PMC137774

[27] Sarasin-Filipowicz M, Oakeley EJ, Duong FH, Christen V, Terracciano L, et al (2008) Interferon signaling and treatment outcome in chronic hepatitis C. Proc Natl Acad Sci U S A 105: 7034–7039. 18467494 10.1073/pnas.0707882105 PMC2383932

[28] Chen J, Young NL, Subbarao S, Warachit P, Saguanwongse S, et al (1999) HIV type 1 subtypes in Guangxi Province, China, 1996. AIDS Res Hum Retroviruses 15: 81–84. 10024057 10.1089/088922299311754

[29] Feld JJ, Nanda S, Huang Y, Chen W, Cam M, et al (2007) Hepatic gene expression during treatment with peginterferon and ribavirin: Identifying molecular pathways for treatment response. Hepatology 46: 1548–1563. 17929300 10.1002/hep.21853 PMC2808168

[30] Asselah T, Bieche I, Sabbagh A, Bedossa P, Moreau R, et al (2009) Gene expression and hepatitis C virus infection. Gut 58: 846–858. 19074178 10.1136/gut.2008.166348 PMC2673514

[31] Li K, Li NL, Wei D, Pfeffer SR, Fan M, et al (2012) Activation of chemokine and inflammatory cytokine response in hepatitis C virus-infected hepatocytes depends on Toll-like receptor 3 sensing of hepatitis C virus double-stranded RNA intermediates. Hepatology 55: 666–675. 22030901 10.1002/hep.24763 PMC3272326

[32] Wang N, Liang Y, Devaraj S, Wang J, Lemon SM, et al (2009) Toll-like receptor 3 mediates establishment of an antiviral state against hepatitis C virus in hepatoma cells. J Virol 83: 9824–9834. 19625408 10.1128/JVI.01125-09 PMC2747996

[33] Saito T, Owen DM, Jiang F, Marcotrigiano J, Gale M Jr (2008) Innate immunity induced by composition-dependent RIG-I recognition of hepatitis C virus RNA. Nature 454: 523–527. 18548002 10.1038/nature07106 PMC2856441

[34] Arnaud N, Dabo S, Akazawa D, Fukasawa M, Shinkai-Ouchi F, et al (2011) Hepatitis C virus reveals a novel early control in acute immune response. PLoS Pathog 7: e1002289. 22022264 10.1371/journal.ppat.1002289 PMC3192838

[35] Han JQ, Barton DJ (2002) Activation and evasion of the antiviral 2'-5' oligoadenylate synthetase/ribonuclease L pathway by hepatitis C virus mRNA. RNA 8: 512–525. 11991644 10.1017/s1355838202020617 PMC1370272

[36] Zhou Z, Wang N, Woodson SE, Dong Q, Wang J, et al (2011) Antiviral activities of ISG20 in positive-strand RNA virus infections. Virology 409: 175–188. 21036379 10.1016/j.virol.2010.10.008 PMC3018280

[37] Strader DB, Wright T, Thomas DL, Seeff LB (2004) Diagnosis, management, and treatment of hepatitis C. Hepatology 39: 1147–1171. 15057920 10.1002/hep.20119

[38] Levy DE, Darnell JEJ (1990) Interferon-dependent transcriptional activation: Signal transduction without second messenger involvement. New Biol 2: 923–928. 1706625

[39] Pine R, Canova A, Schindler C (1994) Tyrosine phosphorylated p91 binds to a single element in the ISGF2/IRF-1 promoter to mediate induction by IFNa and IFNg, and is likely to autoregulate the p91 gene. EMBO J 13: 158–167. 8306959 10.1002/j.1460-2075.1994.tb06245.x PMC394789

[40] Park H, Serti E, Eke O, Muchmore B, Prokunina-Olsson L, et al (2012) IL-29 is the dominant type III interferon produced by hepatocytes during acute hepatitis C virus infection. Hepatology 56: 2060–2070. 22706965 10.1002/hep.25897 PMC3581145

[41] Thomas E, Gonzalez VD, Li Q, Modi AA, Chen W, et al (2012) HCV infection induces a unique hepatic innate immune response associated with robust production of type III interferons. Gastroenterology 142: 978–988. 22248663 10.1053/j.gastro.2011.12.055 PMC3435150

[42] Honda M, Sakai A, Yamashita T, Nakamoto Y, Mizukoshi E, et al (2010) Hepatic ISG expression is associated with genetic variation in interleukin 28B and the outcome of IFN therapy for chronic hepatitis C. Gastroenterology 139: 499–509. 20434452 10.1053/j.gastro.2010.04.049

[43] Lanford RE, Guerra B, Bigger CB, Lee H, Chavez D, et al (2007) Lack of response to exogenous interferon-alpha in the liver of chimpanzees chronically infected with hepatitis C virus. Hepatology 46: 999–1008. 17668868 10.1002/hep.21776 PMC2386986

[44] Li K, Foy E, Ferreon JC, Nakamura M, Ferreon AC, et al (2005) Immune evasion by hepatitis C virus NS3/4A protease-mediated cleavage of the Toll-like receptor 3 adaptor protein TRIF. Proc Natl Acad Sci U S A 102: 2992–2997. 15710891 10.1073/pnas.0408824102 PMC548795

[45] Meylan E, Curran J, Hofmann K, Moradpour D, Binder M, et al (2005) Cardif is an adaptor protein in the RIG-I antiviral pathway and is targeted by hepatitis C virus. Nature 437: 1167–1172. 16177806 10.1038/nature04193

[46] Li XD, Sun L, Seth RB, Pineda G, Chen ZJ (2005) Hepatitis C virus protease NS3/4A cleaves mitochondrial antiviral signaling protein off the mitochondria to evade innate immunity. Proc Natl Acad Sci U S A 102: 17717–17722. 16301520 10.1073/pnas.0508531102 PMC1308909

[47] Heim MH (2013) Innate immunity and HCV. J Hepatol 58: 564–574. 23063572 10.1016/j.jhep.2012.10.005

[48] Garaigorta U, Chisari FV (2009) Hepatitis C virus blocks interferon effector function by inducing protein kinase R phosphorylation. Cell Host Microbe 6: 513–522. 20006840 10.1016/j.chom.2009.11.004 PMC2905238

[49] Berres ML, Koenen RR, Rueland A, Zaldivar MM, Heinrichs D, et al (2010) Antagonism of the chemokine Ccl5 ameliorates experimental liver fibrosis in mice. The Journal of clinical investigation 120: 4129–4140. 20978355 10.1172/JCI41732 PMC2964968

[50] Katsounas A, Trippler M, Kottilil S, Lempicki RA, Gerken G, et al (2012) Cytokine/chemokine patterns connect host and viral characteristics with clinics during chronic hepatitis C. Eur J Med Res 17: 9. 22577869 10.1186/2047-783X-17-9 PMC3489717

[51] Schulze-Krebs A, Preimel D, Popov Y, Bartenschlager R, Lohmann V, et al (2005) Hepatitis C virus-replicating hepatocytes induce fibrogenic activation of hepatic stellate cells. Gastroenterology 129: 246–258. 16012951 10.1053/j.gastro.2005.03.089

[52] Urban TJ, Thompson AJ, Bradrick SS, Fellay J, Schuppan D, et al (2010) IL28B genotype is associated with differential expression of intrahepatic interferon-stimulated genes in patients with chronic hepatitis C. Hepatology 52: 1888–1896. 20931559 10.1002/hep.23912 PMC3653303

[53] Marukian S, Andrus L, Sheahan TP, Jones CT, Charles ED, et al (2011) Hepatitis C virus induces interferon-lambda and interferon-stimulated genes in primary liver cultures. Hepatology 54: 1913–1923. 21800339 10.1002/hep.24580 PMC3219820

